# South Africa’s Best BARK Medicines Prescribed at the Johannesburg Muthi Markets for Skin, Gut, and Lung Infections: MIC’s and Brine Shrimp Lethality

**DOI:** 10.3390/antibiotics10060681

**Published:** 2021-06-07

**Authors:** Gugulethu P. Khumalo, Nicholas J. Sadgrove, Sandy F. Van Vuuren, Ben-Erik Van Wyk

**Affiliations:** 1 Department of Botany and Plant Biotechnology, University of Johannesburg, P.O. Box 524, Auckland Park 2006, South Africa; bevanwyk@uj.ac.za; 2 Jodrell Science Laboratory, Royal Botanic Gardens Kew, Richmond, Surrey TW9 3DS, UK; 3 Department of Pharmacy and Pharmacology, Faculty of Health Sciences, University of the Witwatersrand, 7 York Road, Park Town 2193, South Africa; sandy.vanvuuren@wits.ac.za

**Keywords:** bark, antibacterial, traditional medicine, Zulu, brine shrimp lethality

## Abstract

Indigenous trade of medicinal plants in South Africa is a multi-million-rand industry and is still highly relevant in terms of primary health care. The purpose of this study was to identify today’s most traded medicinal barks, traditionally and contemporaneously used for dermatological, gastrointestinal, and respiratory tract infections; then, to investigate the antimicrobial activity and toxicity of the respective extracts and interpret outcomes in light of pharmacokinetics. Thirty-one popularly traded medicinal barks were purchased from the Faraday and Kwa Mai-Mai markets in Johannesburg, South Africa. Information on the medicinal uses of bark-based medicines in modern commerce was recorded from randomly selected traders. The minimum inhibitory concentration (MIC) method was used for antimicrobial screening, and brine shrimp lethality was used to determine toxicity. New medicinal uses were recorded for 14 bark species. Plants demonstrating some broad-spectrum activities against tested bacteria include *Elaeodendron transvaalense*, *Erythrina lysistemon*, *Garcinia livingstonei*, *Pterocelastrus rostratus*, *Rapanea melanophloeos*, *Schotia brachypetala*, *Sclerocarya birrea*, and *Ziziphus mucronata*. The lowest MIC value of 0.004 mg/mL was observed against *Staphylococcus epidermidis* for a dichloromethane bark extract of *E. lysistemon*. The tested medicinal barks were shown to be non-toxic against the *Artemia nauplii* (brine shrimp) bioassay, except for a methanol extract from *Trichilia emetica* (69.52% mortality). Bacterial inhibition of bark extracts with minimal associated toxicity is consistent with the safety and valuable use of medicinal barks for local muthi market customers. Antimicrobial outcomes against skin and gastrointestinal pathogens are feasible because mere contact-inhibition is required in vivo; however, MIC values against respiratory pathogens require further explaining from a pharmacokinetics or pharmacodynamics perspective, particularly for ingested rather than smoked therapies.

## 1. Introduction

Bark is the outer protective layer of woody plants. It consists of all tissues outside the vascular cambium, an actively dividing layer of cells responsible for the production of xylem and phloem tissues [1,2]. Bark contains a high mass of polyphenols with medicinal applications [3,4]. Biological studies of medical bark remain scarce, which has led to it being undervalued as a suitable organ [3]. The use of bark in traditional medicine is also common in other parts of the world outside of Africa. A study by Pagliosa et al. [5] conducted in Brazil reported potent antioxidant activity from the methanol extract of bark from *Ilex paraguariensis* A. St. Hil. Furthermore, medicinal bark of different plant species from the British Moracin family were reported to contain bioactive benzofuran derivatives [6]. From south and southeast Asian countries, species from the genus *Dillenia* are a rich source of flavonoids, flavones, triterpenes, and tannins [7]. In Cameroon, the bark of *Araliopsis soyauxii* Engl., popularly used in traditional medicine, yielded bioactive alkaloids that can be used as anticancer agents [8]. In India, the bark of *Oroxylum indicum* (L.) is commonly used in Ayurvedic medicine; the extracts exhibited anti-adipogenesis activity and pancreatic lipase inhibition [9].

The widespread significance and impact of bark medicines across human cultures is implicit in most of the pharmacopoeias, and encapsulated in commerce and industry, which boasts several well-known effective bark-sourced drugs. Quinine, for example, is an anti-malarial alkaloid isolated from the bark of the South American species *Cinchona officinalis* L. and other species [10]. Paclitaxel (Taxol) is a widely used cancer drug, originally derived from the bark of *Taxus brevifolia* Nutt. [11]. Aspirin (acetylsalicylic acid) is derived from salicin, a precursor from the bark of willow (*Salix* spp.) [12].

Despite the importance of bark in traditional medicine, there are relatively few studies focusing on the biological screening of bark. In southern Africa, bark is the most frequently used plant part in the preparation of herbal remedies, and accounts for one-third of plant material traded in South African informal traditional markets [13,14,15,16,17]. The trade of indigenous plants in South Africa is part of a multi-million-rand industry [18]. Of the estimated 2062 indigenous medicinal plant species in South Africa, 37% are sold on muthi markets in KwaZulu-Natal, Gauteng, Eastern Cape, Mpumalanga and the Limpopo Province [19]. From the 771 plant species traded, the woodland and forest biomes contribute about 574 plant products [20]. Bark and root materials are the dominant plant parts traded in traditional markets of South Africa because of their long-term storage ability [15,21].

In Johannesburg, the two major trading centers for traditional medicine are the Kwa Mai Mai (14 Berea Rd, City and Suburban, Johannesburg, 2094, South Africa) and Faraday (Salisbury Claims, Johannesburg, 2001, South Africa) Muthi Markets [22]. Visitors to the markets consult with a medical herbalist and receive recommendations [23]. They may also have been sent to the markets by a sangoma, a spiritual healer that uses divinatory methods, or an inyanga, a sophisticated herbalist that communicates with their deceased ancestors before making herbal prescriptions [24]. While the sangoma is often consulted to enact magical effects, such as love spells and so forth, the inyanga is more often consulted for general health afflictions. Because the most common maladies afflicting visitors of the markets are respiratory, skin, and gut afflictions, the inyanga or muthi market trader is the most consulted in these contexts.

The widespread and long-term use of traditional medicinal plants as antibacterial agents gives the impression that they are safe [25,26]. However, studies have shown that many plants used as food or traditional medicine are toxic [26,27,28]. Toxicity studies of southern African medicinal plants traditionally used against infectious diseases have recently gained popularity [27,28,29,30,31]. Most toxicity assays of plant extracts have hitherto focused on prospecting for compounds that are cytotoxic against cancerous cells rather than normal human cells [30]. Scientific evaluation of toxic properties of traditional remedies is of major public concern, as large populations in developing countries depend on or prefer the use of traditional medicines. This study aims to determine antimicrobial activity and toxicity of 31 popularly traded medicinal barks used against dermatological, gastrointestinal, and respiratory tract infections.

## 2. Results and Discussion

### 2.1. Muthi Market Survey

The use of traditional medicine is strongly based on cultural and spiritual beliefs and is not limited to the rural population, as these plant products are also found in urban areas and widely purchased or used by educated individuals [32,33]. A survey that was carried out in 2001 at the Faraday Muthi Market revealed that each year, approximately 12–15 million consumers from urban settlements depend on traditional medicine derived from woodlands and forests [17].

Eighty-five percent of traders were Zulu speaking, and 14% were Xitsonga and Xhosa speakers each (but both communicated in Zulu). Therefore, communication, recording, and writing of the Zulu vernacular names and other information provided was accurately done. In South Africa, informal trading of medicinal plants is very popular among black populations. Similar results were reported by Williams [34], who stated that nearly all the traders at the Faraday Muthi Market are Zulu speaking. The percentage of languages of the traders recorded by Williams [35] were 95.8% Zulu, 2.1% Sotho, 1.1% Xhosa, and 1.1% Tsonga. The majority of traders (traditional healers in particular) are from KwaZu-lu-Natal (the Zululand region), as they mentioned that they harvest the plant materials from kwaNongoma, Bergville, and kwa-Mhlab’ uyalingana area. Language data recorded in this study clearly show that the Faraday and Kwa Mai-Mai Muthi Market traders are all South Africans, with the majority of them coming from the KwaZulu-Natal Province. In 1998, there was already an estimate of six million people in the KwaZulu-Natal Province who were involved in the indigenous trade of medicinal plants, either as harvesters, sellers, or buyers [32,36].

Observations from our study confirmed that very few, if any, traders are from other South African cultures, and none were recorded from other southern African countries. In contrast, Mander et al. [15] reported that many traders from the street markets of Dur-ban and Johannesburg come from Mozambique and Swaziland due to the active plant trade from these countries to South Africa. Our observations agree with the data of Williams [17] who reported that 97% of the approximately 166 traders are migrants to the Faraday Muthi Market, of whom 90% regarded the province of KwaZulu-Natal as “home”.

Gender information of traders at the Johannesburg Muthi Markets revealed that 64% of the traders are females and 36% males. Results from the present study are in accordance with Williams [17], who reported that 70% of the Faraday Muthi Market traders in Johannesburg were women and only 30% were males. Another survey on muthi markets in Zululand reported that out of 63 plant traders interviewed, 77% were females [37]. A report by Mander et al. [15] indicated that an estimated 74% of medicinal plant harvesters, street traders, and traditional healers are women, and 80% are rural and 20% urban. Twenty-six percent of the trade role players are men, of which 50% are rural and 50% urban. Thus, women are the main role players in the medicinal plant trade, predominantly those from rural areas. Their involvement in the medicinal plant trade is regarded as an important source of income, unlike males, who are mostly employed in formal job sectors [15,17,37].

Of the ca. 70 medicinal barks that are commonly sold at the Johannesburg Muthi Markets [23], 31 species belonging to 19 families are used against dermatological, gastrointestinal, and respiratory tract infections. The leading families of the selected 31 species are Fabaceae (37%), Anacardiaceae (16%), Lauraceae (16%), and Celastraceae (10%). The Euphorbiaceae, Anacardiaceae, and Celastraceae were found to be the top three families in a review by Grace et al. [13], which listed 174 bark species from 51 families that are used in traditional medicine in the KwaZulu-Natal province. An ethnobotanical survey conducted in the northern Maputaland, KwaZulu-Natal province showed that most of the plants used for skin ailments by the rural inhabitants belong to the family Fabaceae [38].

Traders are very knowledgeable about the plant material they sell, as it is part of their daily activity. In a rare event where there was uncertainty about a sample, it turned out that it was due to similar vernacular names for different plant species or a consistent error in the literature. For example, *Balanites maughamii* Sprague has consistently been referred to in the literature as *ugobandlovu* [13,14,39,40,41] while this name should correctly be applied to *Garcinia livingstonei* T. Anderson.

All the traders on the Johannesburg Muthi Markets insisted that this is the correct application of the vernacular name. Traders on the Durban Muthi Market confirmed that *ugobandlovu* is the correct name for *G. livingstonei* and not *B. maughamii* (B.-E. van Wyk, pers. comm.). The current name for the latter is iphamba. Another example is *umvangazi*, which has sometimes been used in the literature for *Albizia versicolor* [17,39] while it actually refers to *Pter-ocarpus angolensis*. It is therefore clear that there is still a need for systematic documentation of the basic characters and character states of bark even though DNA barcoding and metabolomics methods may be convenient and accurate methods to confirm and verify identifications.

Commercial medicinal uses of 31 barks purchased from the Johannesburg Muthi Markets are presented in Table 1. Medicinal uses recorded from the traders were similar to those recorded from the literature. In some cases, new medicinal plant uses were recorded, and these are shown in bold in Table 1. New uses were recorded for the first time for 14 species (*Albizia adianthifolia*, *Bersama lucens*, *Croton sylvaticus*, *Cryptocarya latifolia*, *Dombeya rotundifolia*, *Elaeodendron transvaalense*, *Erythrina lysistemon*, *Harpephyllum caffrum*, *Prunus africana*, *Schotia brachypetala*, *Syzygium cordatum*, *Trichilia emetica*, *Vachellia natalitia*, and *Vachellia robusta*). Medicinal barks are packaged in small A4 size plastic bags or wrapped in old newspapers. These medicinal barks are used by customers to prepare infusions, decoctions, powders, or pastes. Decoctions and infusions are often used as emetics (“ukhuphalaza”) and enemas (“ukucha-tha”) for treatment of internal disorders, chest complaints, and sometimes used as a wash (“ukugeza”) for treating skin disorders. Powdered bark material is often licked from the palm of the hand (“khotha”) or taken as snuff (“umbemiso”) for treating ailments such as headache, food poisoning, and stroke. Finely powdered bark may also be incorporated into complex mixtures for use as “insizi” (powdered mixtures that are rubbed into scarification in the skin to alleviate pain).

The present study showed that plant species are often used for the treatment of more than one ailment. This has also been reported from various surveys across the country [13,14,42,43]. Mixtures of species are sometimes used as a combination, such as the bark of *Harpephyllum caffrum*, *Schotia brachypetala*, and *Syzygium cordatum*, taken as an emetic to treat respiratory complaints.

**Figure 1:**
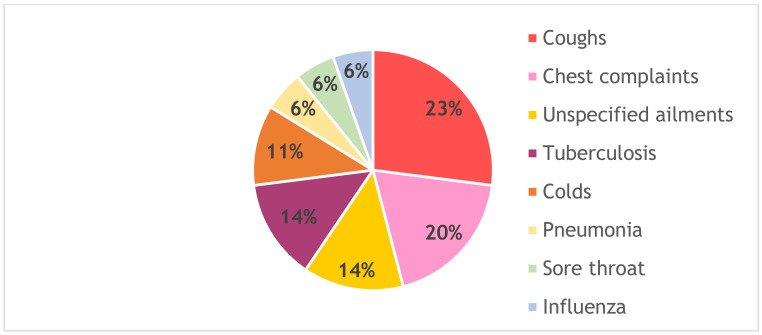
Different ailments of the respiratory tract for which bark is typically used.

The respiratory system is the most vulnerable site for bacterial infections. Environmental instabilities and frequent antimicrobial use are reportedly among the major contributors to bacterial accumulation in the respiratory tract [44]. Results from the present study (Figure 1) reveal that most of the studied medicinal barks are used for the treatment of coughs (23%), chest complaints (20%), tuberculosis (14%), and unspecified respiratory ailments (14%).

Most of the South African population, from rural areas in particular, depend on traditional medicinal plants for the treatment of respiratory ailments [45]. McGaw et al. [46] documented ca. 180 medicinal plants used for treatment of tuberculosis and related symptoms such as chronic cough, respiratory complaints, and fever. In South Africa, infections of the respiratory tract associated with high mortality rates include tuberculosis, pneumonia, and influenza [47,48]. While there are multiple species that are successful in traditional healthcare at treating these bacterial infections, it is still necessary to increase our understanding of the primary mechanisms and magnitude of effects [49].

**Figure 2:**
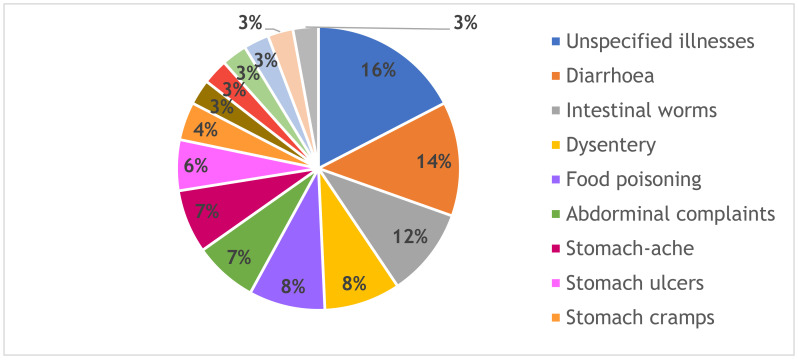
Different ailments of the gastrointestinal tract for which bark is typically used.

Different ailments of the gastrointestinal tract that are commonly treated with bark are shown in Figure 2. Most of the medicinal barks are used against diarrhea (14%), intestinal worms (12%), dysentery (8%), and food poisoning (8%). These findings are in accordance with those reported by other authors [16,50,51] who found that lay people through ethnobotanical surveys use mostly bark of trees for treating diarrhea. In 2005, there were more than 25,000 deaths caused by diarrheal diseases in South Africa [52]. Medicinal plants used traditionally for the treatment of diarrhea are reported to have antispasmodic properties, delay gastrointestinal transit, suppress gut motility, stimulate water adsorption, and/or reduce electrolyte secretion [53].

However, the main reason for using bark against diarrhea is the presence of tannins, which are known to be non-specific protein poisons [34,54]. According to Hutchings [55], tannins are frequently present in those parts of plants used in the treatment of dysentery and diarrhea, or for respiratory ailments. The tannins form a protective, impermeable layer over mucosa and also prevent the development of bacteria by denaturing the proteins within these unicellular organisms. Moreover, of importance is the use of bark against intestinal worms but in this case, the efficacy probably depends on the chemical compounds with specific activity against parasites. Other authors [13,51] also reported the frequent use of bark against internal parasites.

Gastrointestinal infections are predominantly associated with pathogens transmitted from fecal-contaminated water, food, or environment, as well as poor hygiene associated with poverty and a poor nutritional status [56,57]. South African rural communities, including northern Maputaland (KwaZulu-Natal province) and Bizana (Eastern Cape province), have been reported to still lack access to safe drinking water, good hygiene, and sanitation [50,58]. Therefore, antimicrobial studies of traditional medicinal plants used for the treatment of gastrointestinal tract ailments are still very relevant in South Africa.

**Figure 3:**
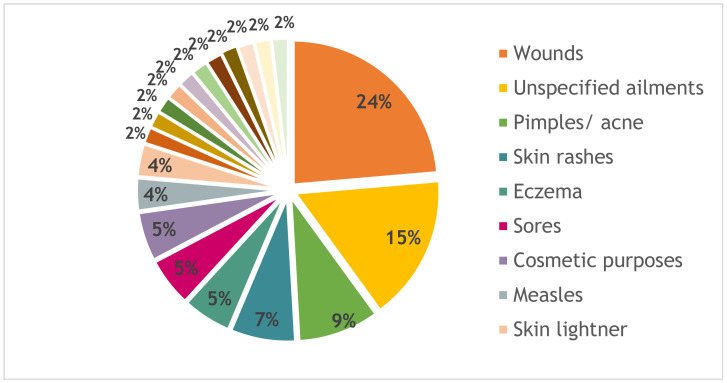
Different skin ailments for which bark is typically used to treat.

Skin ailments and the cosmetic use of bark are shown in Figure 3. Many barks are used to promote wound healing (24%), to remove pimples or acne (9%), and to treat skin rashes (7%). De Wet et al. [38] found that leaves were mostly used for treatment of general skin ailments (31%), closely followed by bark with 28%, and then the roots with 13%.

Grace et al. [13] also found that wound healing is among the most common external applications of bark. The Fabaceae are especially popular for treating wounds [38], as is also shown in the present study.

### 2.2. Antimicrobial Outcomes

A new criterion of interpreting MIC values of medicinal plant extracts stipulates that activity is “noteworthy to moderate” if the MIC values are between 0.1 to 0.6 mg/mL [45]. The antimicrobial activity of methanol and dichloromethane bark extracts tested against pathogens associated with dermatological, gastrointestinal, and respiratory tract infections isshown in Table 2. The MIC values ranged from 0.004 mg/mL to above 8 mg/mL. Considering that MIC values below 1 mg/mL are regarded by some contemporary researchers as qualifying as “active” [59,60], then many of the extracts produced in the current study are active and warrant further investigation. Medicinal barks demonstrating broad-spectrum activities include *Elaeodendron transvaalense*, *Erythrina lysistemon*, *Garcinia livingstonei*, *Pterocelastrus rostratus*, *Rapanea melanophloeos*, *Schotia brachypetala*, *Sclerocarya birrea*, *Warburgia salutaris*, *Prunus africana*, and *Ziziphus mucronata*. The lowest MIC value of 0.004 mg/mL was noted against *S. epidermidis* from the dichloromethane extract of *E. lysistemon*, which is considered acceptable by the strict criteria set forth by another group of authors [61]. *Garcinia livingstonei* and *Kigelia africana* exhibited interestingly low MIC values of 0.06 mg/mL (methanol extract) against *B. cereus* and 0.16 mg/mL for dichloromethane extracts, respectively, which is close to the limits for good activity [59,62]. *Bacillus cereus* was found to be the most susceptible bacterium screened. The antimicrobial activities of medicinal barks seem to support the traditional and contemporary medicinal uses as reported by traders on the Johannesburg Muthi Markets.

#### 2.2.1. *Erythrina lysistemon*

Bark extracts demonstrated interesting antimicrobial activity against the bacterial pathogens screened. Bark of *E. lysistemon* is widely used in traditional medicine for the treatment of skin ailments (Table 1). *Staphylococcus epidermidis* was the most susceptible bacteria against both extracts, yielding the lowest MIC value of 0.12 mg/mL (methanol) and 0.004 mg/mL (dichloromethane). Bacterial inhibition against *S. aureus* (MIC of 0.20 mg/mL) demonstrates the efficacy of its traditional use against wounds. *Staphylococcus aureus* is a bacterial strain associated with wound infection [45,63]. Reports from earlier studies [64] showed that methanol extracts of *E. lysistemon* bark did not exhibit activity against *S. aureus*. The ethyl acetate, ethanol, and water extracts of *E. lysistemon* bark [63] also did not show antimicrobial activity against *S. epidermidis*, *P. aeruginosa*, and *E. coli* on the disc diffusion assay, but activity was only observed against *S. aureus*. The same authors observed that bark of the *Erythrina* species is more active in terms of antibacterial activity than the leaves. The discrepancy in the results could be due to the type of solvent extract used and the different antimicrobial techniques employed. However, more recent results by Mabona et al. [63] demonstrated comparable noteworthy activity from the leaf extract against *S. aureus* with an MIC value of 0.20 mg/mL. In addition, Mukandiwa et al. [65] reported comparable MIC values against *S. aureus* (0.31 mg/mL) from methanol and dichloromethane extracts of *E. lysistemon* leaves.

Antimicrobial screening of bark extracts against the newly reported traditional medicinal use for stomach ailments (Table 1) resulted in broad-spectrum activity against gastrointestinal tract pathogens (Table 2). Noteworthy antimicrobial activity with an MIC value of 0.10 mg/mL (dichloromethane extract) and 0.16 mg/mL (methanol extract) against *E. faecalis* and 0.16 mg/mL for both extracts was noted against *S. sonnei* (Table 2). A follow-up study of these MIC values irrefutably demonstrated that antimicrobial effects were largely attributed to the presence of prenylated isoflavones and pterocarpans. A structure-activity relationships analysis demonstrated that prenylation on rings A and B, with a hydroxyl group on the same ring as the prenyl group, conferred the most potent antimicrobial outcomes [66].

#### 2.2.2. *Garcinia livingstonei*

The methanol extract of *G. livingstonei* demonstrated the best antimicrobial activity against most of the bacteria responsible for gastrointestinal tract infections screened. *Garcinia livingstonei* is widely used for the treatment of diarrhea [13,50]. Noteworthy activity, with MIC values of 0.25 mg/mL against *S. typhimurium* and 0.41 mg/mL against *E. coli* (methanol extracts), was noted. Excellent activity, with the lowest MICs of 0.06 mg/mL against *B. cereus* and 0.18 mg/mL against *E. faecalis*, was noted from the methanol extract. A recent study by Van Vuuren and co-workers in 2015 [67] showed similar results from the organic extract of bark (1:1 methanol: dichloromethane). The reported MIC values were 0.12 mg/mL against *B. cereus*, 0.19 mg/mL against *S. typhimurium*, 0.34 mg/mL against *E. faecalis*, and 0.38 mg/mL against *Shigella flexneri* [67]. Furthermore, isolated chemical compounds from the leaf extract of *G. livingstonei* demonstrated strong activity, with MICs of 0.006 mg/mL and 0.008 mg/mL against *E. faecalis* and *E. coli*, respectively [68,69]. Corresponding biological activity of bark against diarrheal diseases was also reported from *Garcinia buchananii* bark aqueous extract and its fractions, which reversed high-lactose-diet diarrhea-induced weight loss and reduced bloating as well as fecal fluid content on the tested rats [70].

**Table 1:** Traditional medicinal uses of 31 popularly traded barks used against dermatological, gastrointestinal, and respiratory tract infections.

Species	Bark Descriptions	Skin Ailments	Gastrointestinal Ailments	Respiratory Ailments	Other Uses of Bark	References
*Albizia adianthifolia* (Schumach.) W. Wight (Fabaceae)	OUTER BARK APPEARANCE Yellowish brown. Smooth in young stems; rough in mature trunk with irregular cracks. Flakes sometimes appear rectangular. On young stems, lenticels are small to medium-sized, roundish in shape, and scattered. INNER BARK APPEARANCE Yellow (10 YR 7/6) to strong brown (7.5 YR 5/68). Rough and fibrous. Fibers come off as threads.	Pounded bark is used as an aqueous lotion for eczema and other itchy skin complaints, as a body wash, or as a facial sauna. Bark is used in cosmetics, and for skin diseases and scabies. Bark is soaked in hot water and used as a wash for chickenpox	No traditional use	No traditional use	Bark is used for cleansing of blood, bronchitis, gynecological disorders; for epilepsy, gonorrhea, and eye sight problems. Powdered bark is used as a snuff for headaches and sinusitis	[13,14,40,43,71,72,73,74]
*Bersama lucens* (Hochst.) Szyszył. (Melianthaceae)	OUTER BARK APPEARANCE Brownish yellow (green layer underneath). Young stems smooth with tiny vertical fissures; green layer is present underneath periderm. Lenticels scattered, small to medium-sized, and arranged in horizontal (sometimes-vertical) rows. Mature bark is rough with dominant vertical cracks. Scales can be vertical and irregular. INNER BARK APPEARANCE Yellow (2.5 Y 7/6). Smooth and solid.	No traditional use	Bark is boiled and incorporated into herbal mixtures (*imbiza*), used as an emetic for stomach ailments	No traditional use	Bark is used for barrenness and impotence, to relieve menstrual pain and leprosy, and to calm nervous disorders and sexually transmitted infections. Powdered bark is taken as a snuff to treat congestive headaches, strokes, and apoplexy	[13,14,40,43,71,75,76]
*Calodendrum capense* (L. f.) Thunb. (Rutaceae)	OUTER BARK APPEARANCE Brownish grey. Smooth, even in mature trunk, with small shallow vertical cracks. In young stems, a green layer is present underneath the periderm. Lenticels are in vertical rows or scattered. INNER BARK APPEARANCE Very pale yellow (Munsell white diagram 2.5 Y 9/2). Smooth with visible patterns of rays in diagonal lines.	Bark is used as an ointment and moisturizer to remove pimples. Paste is applied facially as a skin lightener	No traditional use	No traditional use	No traditional use	[13,14,40,71,75,77]
*Cinnamomum camphora* (L.) J.Presl * (Lauraceae)	OUTER BARK APPEARANCE Dark reddish brown and light brown in cracks. Bark is rough and deeply furrowed. No lenticels. INNER BARK APPEARANCE Reddish yellow (7.5 YR 6/8). Very smooth (slippery)	Bark is used for skin ailments	Bark is used to relieve abdominal discomfort	Bark is used for colds and influenza	Bark is used as a perfume and to treat fever	[13,14,39,40,71,78]
*Croton sylvaticus* Hochst. (Euphorbiaceae)	OUTER BARK APPEARANCE Yellowish grey to brown. Smooth with green layer underneath. Lenticels small to medium with horizontal or vertical apertures; lenticels scattered or present inside cracks in mature bark samples. INNER BARK APPEARANCE Light yellowish brown (10 YR 6/4) to brownish yellow (10 YR 6/6). Smooth and solid.	Pounded decoction is used for wounds. Finely ground bark is rubbed into incisions on the skin as an irritant for inflammation	Decoction is used for abdominal disorders, digestive and intestinal complaints	The bark is used as a remedy taken orally to treat tuberculosis (TB). Powdered bark is applied topically for chest pain	Decoction is used for rheumatism, fever, inflammation, and uterine diseases	[13,14,39,43,79]
*Cryptocarya latifolia* Sond. (Lauraceae)	OUTER BARK APPEARANCE Brown (7.5 YR, 4/3). Smooth, lenticels small (<1 mm) and scattered. INNER BARK APPEARANCE Very dark brown (7.5 YR, 2.5/3). Smooth, sometimes with small ridges.	No traditional use	Powdered bark infusion is used for stomach cramps	Powdered bark mixed with crocodile fat and is used to treat chest ailments. Bark is also used to treat tuberculosis	Internal pains, allergy. An infusion is used orally for muscular cramps. Decoction is used as enemas for urinary tract diseases, uterine spasms, and menstrual pains	[13,14,43,80]
*Dombeya rotundifolia* (Hochst.) Planch. (Malvaceae)	OUTER BARK APPEARANCE Grey to dark brown. Rough with irregular cracks. INNER BARK APPEARANCE Reddish yellow (5 YR 7/6). Fibrous but smooth. Fibees peel off as long strips.	Decoction is used to steam “*ukufutha*” for itchy skin problems. Inflexible fibers of the bark are used to cover wounds.	Bark or wood infusions are taken orally or as enemas for intestinal ulcers, stomach complaints, and diarrhea	No traditional use	Bark is mixed with banana seeds (*Momordica balsamina*) to treat heart diseases. Bark is used for headache, palpitations, nausea, abortion, and irregular menstruation	[13,14,40,43,71,81]
*Ekebergia capensis* Sparrm. (Meliaceae)	OUTER BARK APPEARANCE Light grey with yellowish lenticels in young stems. Grey-brown in mature stems. Bark is relatively smooth in young stems with tiny vertical fissures; lenticels are in vertical rows 2 to 20 mm apart. Bark is rough in mature trunk with irregular cracks. INNER BARK APPEARANCE Strong brown (7.5 YR 5/8; 7.5 YR 4/4). Relatively smooth. Looks fibrous, but solid.	Ground bark is mixed with flour in water to form a poultice to treat abscesses and boils. Hot infusion is used as a wash for pimples	Powdered bark and roots are mixed to make tea in order to treat gastritis	Decoction is taken as emetics for respiratory and chest complaints	Bark is used to treat malaria, heartburn, and sexually transmitted diseases. Infusion is taken as an emetic for purifying blood	[13,14,39,40,43,71,82]
*Elaeodendron transvaalensis* (Burtt Davy) R. H. Archer (Celastraceae)	OUTER BARK APPEARANCE Grey with prominent orange color underneath the periderm. Bark is relatively smooth with vertical fissures. Lenticels are not visible. INNER BARK APPEARANCE Pinkish white (5 YR 8/2). Smooth and solid.	The bark is used for skin rashes and skin infections	Decoction is taken orally or as an enema for stomach complaints and internal wounds. Decoction and/or powdered bark is licked to treat diarrhea, intestinal worms, and stomach cramps	No traditional use	Bark is used to treat fever, piles, kidney, and bladder infections; to relieve body pain and for heavy menstruation	[13,14,40,43,71,78,83,84]
*Erythrina lysistemon* Hutch. (Fabaceae)	OUTER BARK APPEARANCE Grey brown, green underneath the superficial layer (in very old trunks greenish yellow). Relatively smooth with longitudinal grooves and scattered knob-like projections with hooked pointed thorns. Lenticels are prominent in vertical rows. INNER BARK APPEARANCE Yellow (10 YR 7/8). Smooth with visible rays.	The bark is used as a poultice for swellings and abscesses. Ash from burnt bark is used to disinfect wounds	Infusion is used for internal wounds, allergies, and stomach ulcers, and used as an emetic for food poisoning	No traditional use	Bark is used to facilitate childbirth or taken as tea to relieve labor pains, for toothache, and as a mouthwash	[13,14,40,43,71,73,85,86]
*Erythrophleum lasianthum* Corbishley (Fabaceae)	OUTER BARK APPEARANCE Bark is grey on the superficial part and becomes strong brown underneath and is dark red further down. Smooth in young stems and rough with irregular (mostly vertical) cracks in mature trunk. INNER BARK APPEARANCE The bark may be grey or dark brown. Smooth with visible stripes that are vertical or sometimes twisted.	No traditional use	Decoction is taken for intestinal spasms, abdominal pains, and as a strong purgative and an anthelmintic	Powdered bark is used for colds	Powdered bark is snuffed to relieve headache. Bark is used in ethnoveterinary medicine	[13,14,40,43,71]
*Garcinia livingstonei* T. Anderson (Clusiaceae)	OUTER BARK APPEARANCE Grey, but orange underneath superficial layer. Smooth in younger stems. Rough, with prominent vertical cracks in mature stem. The bark resembles a mosaic appearance. INNER BARK APPEARANCE Yellowish red (5 YR 5/8). Relatively smooth, saturated with many small dots (exudates) that are dark reddish in color.	The bark is used for cosmetics purposes as a skin lightener	Bark is used for treatment of internal parasites and diarrhea	Stem is used to treat coughs	Bark is used to treat fever	[13,14,39,50,87]
*Harpephyllum caffrum* Bernh. (Anacardiaceae)	OUTER BARK APPEARANCE Brownish in young stems and grey in mature bark. Young stems smooth with vertical fissures and scattered lenticels. Bark from mature stem is rough with dominant vertical cracks forming rectangular to irregular scales. INNER BARK APPEARANCE Red and smooth.	Decoction is used as a wash for skin ailments such as acne and eczema. The bark is used to purify the skin for cosmetic purposes by means of facial steam bath and to reduce pimples	Powdered bark is used licked “*khotha*” for treatment of food poisoning	The bark of *H. caffrum* is mixed with bark of *Schotia brachypetala* and *Syzygium cordatum* and ground into powder, boiled, and taken as an emetic to treat respiratory complaints	Powdered burnt bark is rubbed into scarification, around sprains and fractures. Bark is used for diabetes; decoctions are taken as emetics to purify blood and strengthen the body	[13,14,39,40,43,71,77,78]
*Kigelia africana* (Lam.) Benth. (Bignoniaceae)	OUTER BARK APPEARANCE Greyish brown. Relatively smooth with vertical fissures, no visible lenticels. Mature trunk can be with rectangular or irregular scales. INNER BARK APPEARANCE Dark brown (7.5 YR 3/3). Smooth, sometimes with visible rays.	Stem and twigs are used to treat sores, wounds, and snakebites	Ground bark decoctions are administered as enemas for stomach ailments in children. The bark is used for ulcers while the stem and twigs are used for dysentery	Bark is used to treat pneumonia	Bark is used for syphilis, Human Immunodeficiency Virus (HIV), gonorrhea, and rheumatism. Decoction is used as a mouthwash to relieve pain and inflammation caused by toothache	[13,14,40,71,72,88]
*Ocotea bullata* (Burch.) E. Mey. in Drége (Lauraceae)	OUTER BARK APPEARANCE Grey to brown. Relatively smooth and even in mature trunks. Roundish or slightly elongated dark lenticels scattered on young stems. INNER BARK APPEARANCE Brown (7.5 YR 4/4) to dark brown (7.5 YR 3/2). Relatively smooth with vertical ridges.	No traditional use	The bark is used as a general tonic and for diarrhea in children. Bark is also used for a “bleeding stomach”	No traditional use	Bark is used for nervous disorders. Powdered bark is mixed with *Croton gratissimus* and *Zingiber officinale* root for urinary complaints. Bark powder is snuffed or smoked for headache	[13,14,39,40,43,71]
*Peltophorum africanum* Sond. (Fabaceae)	OUTER BARK APPEARANCE Light grey to black and becomes strong brown and yellowish underneath. Smooth in young stems, rough with vertical cracks and horizontal fissures in medium-sized stems, and very rough with irregular cracks in old mature trunks. Lenticels are present on smooth parts and their remains. INNER BARK APPEARANCE Brown (7.5 YR 4/6) to red (2.5 YR 3/6). Fibrous, fibres flexible when wet.	Decoction is used to treat wounds	Decoction is used to treat diarrhea, dysentery, intestinal parasites, and ulcers, and as a general tonic. Fresh bark is chewed to treat colic	Bark is used for coughs, sore throats, and tuberculosis	Bark and roots are used for sterility, backache, and sexually transmitted infections	[13,14,39,42,78,89]
*Pittosporum viridiflorum* Sims (Pittosporaceae)	OUTER BARK APPEARANCE Greyish brown. Relatively smooth. Lenticels small to medium-sized with horizontal apertures scattered or in distinct horizontal rows. INNER BARK APPEARANCE Pale brown (10 YR 6/3). Very smooth and solid.	No traditional use	Decoction is used as emetics or enemas for stomach problems	Bark is used to treat chest complaints	Decoction is used for malaria and cancer, to induce febrile complaints, and for back pains. Powdered bark is used for toothache. Used as a chewing stick to manage oral fungal infections in HIV patients	[13,14,39,40,71,78,90]
*Prunus africana* (Hook.f.) Kalkman (Rosaceae)	OUTER BARK APPEARANCE Grey to black or dark brown to black. Very rough, with various irregular vertical and horizontal cracks, forming irregular scales. No lenticels. INNER BARK APPEARANCE Dark red (2.5 YR 3/6) or strong brown (7.5 YR 4/6). Fibrous and brittle.	The bark is used for the treatment of allergies and for wound dressing	Bark is used for stomachache	Infusion is administered orally to treat colds and influenza. The bark is used for chest pain	Decoction is used for intercostal pains, urinary tract disorders, kidney diseases, malaria, and inflammation. Bark is active against prostatic hypertrophy	[13,14,39,40,43,71,82,91]
*Pterocelastrus rostratus* *Walp.* (Celastraceae)	OUTER BARK APPEARANCE Greyish brown. Bark is smooth with small scattered yellowish lenticels. INNER BARK APPEARANCE Dark reddish brown (2.5 YR 3/3). Smooth with solid fibrous appearance. Fibers brittle break if pulled.	No traditional use	No traditional use	Decoction is taken as emetics for respiratory ailments, chest block, and wheezing chest	Powdered bark mixed with that of *Rapanea melanophloeos* is added in warm water and taken orally to relieve general body pains	[13,14,43]
*Rapanea melanophloeos* (L.) Mez (Primulaceae)	OUTER BARK APPEARANCE Greyish brown (10 YR 5/2) and reddish towards the inner surface. Relatively smooth with vertical fissures in young and rough with irregular cracks in mature trunk. INNER BARK APPEARANCE Reddish black (5 R 2.5/1). Relatively smooth in touch with visible rays, which is lighter in color than ground tissues. Often vertical cracks occur in samples.	Bark is used as a skin lightener and to treat wounds	Bark is used for acidity and stomach ailments. Decoction is taken for hematemesis and stomachache	Fresh pieces of dried powdered bark are chewed to relieve sore throats	The bark is used for strengthening the heart, and for muscular pains and fever	[13,14,39,40,43,58,71,73]
*Rauvolfia caffra* Sond. (Apocynaceae)	OUTER BARK APPEARANCE Yellow to light brown. Smooth in young stems, rough with short vertical cracks, and horizontal lines on mature trunk. Periderm is spongy to the touch (such as polystyrene). Lenticels are present, even in mature bark from the trunk. They are medium in size and scattered. INNER BARK APPEARANCE Yellowish brown (10 YR 5/6). Relatively smooth and spongy to the touch. Sometimes with visible rays.	Bark is applied topically for measles, urticaria, and other skin rashes. Infusion is used to kill maggots in wounds	Decoction is used for abdominal complaints	The bark is used to treat pneumonia and chewed to relieve coughs	Decoction is used for fever, as a tranquilizer for hysteria, and for insomnia and malaria. It is used for general body swelling, rheumatism, and uterine complaints	[13,14,39,40,42,43,71,73]
*Schotia brachypetala* Sond. (Fabaceae)	OUTER BARK APPEARANCE Grey brown. Smooth in young stems, coarse with vertical or rarely horizontal fissures. No lenticels are visible. INNER BARK APPEARANCE Light red (2.5 YR 6/8). Smooth with brittle fibres.	Infusion is taken as emetics for pimples. The bark is also used topically as a wash and to reduce swellings on the body	Powdered bark is used for food poisoning. The bark is soaked in cold water to make “*ukhamba*” (medication) used as an enema to clean bleeding stomach	Powdered bark of *S. brachypetala* is mixed with bark of *H. caffrum* and *Syzygium cordatum*, put to boil, and used as an emetic to treat respiratory complaints	Decoction is taken for heartburn and a “hangover headache”. Bark is used to treat nervous and cardiac conditions	[13,14,39,40,43,71]
*Sclerocarya birrea* (A. Rich.) Hochst. (Anacardiaceae)	OUTER BARK APPEARANCE Grey to black. Smooth in young stems. Trunk and old branches with irregular or sometimes roundish scales; mature old trunk with strong and prominent vertical cracks. No lenticels present. INNER BARK APPEARANCE Dark reddish brown (5 YR 3/3). Smooth, with papery strips or layers that peel off easily.	Inner bark is boiled and applied as poultice for smallpox, skin ulcers, and skin complaints	Decoctions are administered as enemas for treatment of diarrhea, abdominal complaints, and other stomach ailments	No traditional use	Used for malaria, fever, gonorrhea, headache, toothache, backache, infertility, and to strengthen the heart and to treat diabetes mellitus	[13,14,39,40,42,43,71]
*Securidaca longepedunculata* Fresen. (Polygalaceae)	OUTER BARK APPEARANCE Brown (7.5 YR 4/3). Relatively smooth. Lenticels are present. (Chopped sample) INNER BARK APPEARANCE Yellow (10 YR 8/6) and smooth.	The root or rootbark is used topically for wounds and sores	No traditional use	Rootbark and stembark decoction is used to treat coughs, chest complaints, and tuberculosis	Used for epilepsy. Decoction is used to treat gonorrhea, syphilis, and meningitis	[39,40,71,88]
*Strychnos henningsii* Gilg (Loganiaceae)	OUTER BARK APPEARANCE Greyish brown (lot of lichens, which can make the bark mottled). Bark smooth to rough with very irregular, scattered flakes. Small lenticels scattered or sometimes bigger lenticels in vertically elongated groups. INNER BARK APPEARANCE Yellowish brown (10 YR 5/4). Relatively smooth, sometimes with numerous black marks of mould.	The bark is used to treat schistosomiasis and snakebites	The bark is used as an anthelmintic, a health tonic; to treat schistosomiasis; used as an emetic to treat food poisoning. Bark is also chewed for stomach complaints	No traditional use	Powdered bark is taken with cold water for nausea. Bark is used for malaria, dysmenorrhea, erectile dysfunction, and diabetes mellitus	[13,14,40,43,71,92]
*Syzygium cordatum* Hochst. ex Krauss (Myrtaceae)	OUTER BARK APPEARANCE Grey to dark brown. Smooth in young stems. Mature bark is rough with irregular cracks. No lenticels. INNER BARK APPEARANCE Red (10 R 5/8). Fibrous but smooth. Fibers are flexible when wet.	The bark is used to treat wounds. Bark paste is applied topically for treatment of blisters, pimples, or acne and eczema	The bark is used for internal stomach wounds, diarrhea, and stomach complaints	Powdered bark of *S. cordatum*, *S. brachypetala*, and *H. caffrum* are boiled in water and taken as an emetic to treat respiratory complaints. Decoction is used to treat tuberculosis	Powdered bark is licked (*khotha*) for body pains. Bark is used for headaches, amenorrhoea, and inflammation	[13,14,40,42,71,73,78,93]
*Trichilia emetica* Vahl (Meliaceae)	OUTER BARK APPEARANCE Outer color not useful for identification of this bark, because it can be very mottled due to lichens and moss. Grey-brown, sometimes yellowish. Smooth in young stems. Mature trunk relatively smooth to course, with tiny vertical fissures. No lenticels on mature trunk. INNER BARK APPEARANCE Dark reddish brown (2.5 YR 3/3). Relatively smooth.	Powdered bark is mixed with petroleum jelly and applied on wounds between fingers and toes. Bark is used to promote wound healing	Decoction is used for stomach and intestinal complaints. Infusions are used for dysentery and intestinal worms. Bark infusion of *T. emetica* and *Spirostachys africana* is used for constipation	No traditional use	Bark is used to treat malaria. Infusions are used for rectal ulceration in children	[13,14,39,40,71]
*Vachellia natalitia* (E.Mey.) Kyal. and Boatwr. (Fabaceae)	OUTER BARK APPEARANCE Mottled: light grey in the superficial layer and brown to light brown towards the inner parts. Rough, irregularly fissured, no lenticels. INNER BARK APPEARANCE Yellow (10 YR 8/6). Fibrous (fibers are flexible).	Decoction is taken orally for wounds and is used as an emetic for pimples. Bark is used for cosmetic applications	Ground bark infusion is used for stomachache, diarrhea, and dysentery	Bark is mixed with leaves to make tea for coughs and colds	Infusion is used for hemorrhage, conjunctivitis, and as an antidote for cattle poisoning. Bark and roots are boiled together and used as a mouthwash	[13,14,40,71,73]
*Vachellia robusta* (Burch.) Kyalangalilwa and Boatwright subsp. *robusta* (Fabaceae)	OUTER BARK APPEARANCE Grey to brown. Smooth to rough. Vertical to irregular cracks in old bark. Lenticels only on smooth bark, in horizontal rows. INNER BARK APPEARANCE Light red (2.5 YR 5/8). Fibrous: strong elastic fibers, separated by thin threads. Visible patterns of radial rays.	Bark is used for the treatment of skin disorders	No traditional use	Steam of boiled water from bark is inhaled for chest complaints	Decoction is used for treatment of menstrual pains and sexually transmitted infections	[13,14,40]
*Warburgia salutaris* (G. Bertol.) Chiov. (Canellaceae)	OUTER BARK APPEARANCE Grey to dark brown. Smooth in young branches. Mature bark is rough, brittle with deep irregular cracks. Flakes irregular in shape or vertically elongated. INNER BARK APPEARANCE Pale yellowish pink (7.5 YR -/2). Smooth, sometimes with visible papery layers or may look fibrous, but the fibery layers are very brittle.	Powdered bark is mixed with fats and applied on skin to treat sores and skin eruptions	Purgatives and for stomach ulcers. Bark is used for constipation, stomachache, and other gastrointestinal disorders	Powdered bark is taken with cold water or a pinch; is smoked, sometimes mixed with *Cannabis sativa* leaf to treat colds and a dry cough. Bark is used for chest complaints	Used for malaria, toothache, transmitted infections. Emetic is used for febrile complaints and for rheumatism. Powdered bark is mixed with fats and applied topically for inflammation	[13,14,39,40,42,43,71]
*Ziziphus mucronata* Willd. (Rhamnaceae)	OUTER BARK APPEARANCE Grey to brown or dark brown. Very rough, with very prominent vertical cracks, and some horizontal but less prominent cracks. Mature bark with irregular cracks. INNER BARK APPEARANCE Yellowish red (5 YR 5/8). Smooth with vertical lines.	The bark is used to treat boils, skin infections, and measles. Steam baths from the bark are used to purify skin texture	The bark is used to treat dysentery and other stomach ailments	Infusion is taken as emetics for respiratory ailments and chronic coughs	Bark is used to treat tubercular gland swelling. Decoction is used for rheumatism, gonorrhea, and chlamydia	[13,14,40,51,71]

**Table 2:** Antimicrobial activity of medicinal bark extracts tested against skin, stomach, and chest pathogens (MIC values in mg/mL). Organisms are *Bacillus cereus*, *Staphylococcus aureus*, *Staphylococcus epidermidis*, *Enterococcus faecalis*, *Pseudomonas aeruginosa*, *Escherichia coli*, *Salmonella typhimurium*, *Shigella sonnei*, *Klebsiella pneumoniae*, and *Moraxella catarrhalis*. Bold numbers represent values lower than 1 mg/mL.

Bark Samples	*B. cereus* ATCC 11175		*S. aureus* ATCC 25923		*S. epidermidis* ATCC 12228		*E. faecalis* ATCC 29121		*P. aeruginosa* ATCC 27853		*E. coli* ATCC 8739		*S. typhimurium* ATCC 14028		*S. sonnei* ATCC 9290		*K. pneumoniae* ATCC 13883		*M. catarrhalis* ATCC 23246	
Solvent extracts	M *	DCM **	M	DCM	M	DCM	M	DCM	M	DCM	M	DCM	M	DCM	M	DCM	M	DCM	M	D
*A. adianthifolia*	NT ***	NT	4.00	3.33	4.00	1.33	NT	NT	2.66	2.66	NT	NT	NT	NT	NT	NT	NT	NT	NT	NT
*B. lucens*	**0.33**	**0.50**	NT	NT	NT	NT	**0.50**	**0.50**	NT	NT	2.00	1.00	**0.66**	1.33	1.33	2.00	NT	NT	NT	NT
*C. capense*	NT	NT	5.33	5.33	4.00	4.00	NT	NT	4.00	2.66	NT	NT	NT	NT	NT	NT	NT	NT	NT	NT
*C. camphora*	NT	NT	1.00	1.00	NT	NT	NT	NT	1.66	2.66	NT	NT	NT	NT	NT	NT	1.66	2.00	1.33	2.00
*C. sylvaticus*	0.75	0.50	NT	NT	NT	NT	2.00	1.00	NT	NT	4.00	1.00	2.00	1.66	8.00	3.00	NT	NT	NT	NT
*C. latifolia*	NT	NT	1.66	**0.83**	NT	NT	NT	NT	2.00	1.00	NT	NT	NT	NT	NT	NT	1.66	1.66	2.00	0.83
*D. rotundifolia*	1.33	**0.50**	NT	NT	NT	NT	**0.75**	**0.66**	NT	NT	1.50	1.66	1.00	1.00	2.00	2.66	NT	NT	NT	NT
*E. capensis*	**0.25**	**0.50**	2.00	5.33	2.00	4.00	2.00	2.00	1.00	1.00	1.00	8.00	2.00	2.00	1.00	2.66	NT	NT	NT	NT
*E. transvaalense*	**0.41**	**0.20**	1.66	**0.33**	1.66	**0.26**	**0.50**	**0.26**	**0.83**	**0.37**	**0.66**	**0.41**	1.33	1.00	1.00	**0.75**	NT	NT	NT	NT
*E. lysistemon*	**0.20**	**0.12**	**0.20**	**0.20**	**0.12**	**0.004**	**0.16**	**0.10**	**0.50**	**0.50**	1.00	**0.41**	1.00	1.00	**0.16**	**0.16**	NT	NT	NT	NT
*E. lasianthum*	1.33	**0.83**	NT	NT	NT	NT	1.66	1.33	NT	NT	1.00	2.00	**0.83**	3.00	1.66	8.00	NT	NT	NT	NT
*G. livingstonei*	**0.06**	**0.12**	NT	NT	NT	NT	**0.18**	**0.75**	NT	NT	**0.41**	2.00	**0.25**	2.00	**0.83**	**0.50**	NT	NT	NT	NT
*H. caffrum*	NT	NT	3.33	4.00	1.00	1.00	NT	NT	**0.66**	1.66	NT	NT	NT	NT	NT	NT	NT	NT	NT	NT
*K. africana*	1.66	**0.16**	NT	NT	NT	NT	2.66	**0.83**	NT	NT	1.50	1.33	2.00	2.66	4.00	1.66	NT	NT	NT	NT
*O. bullata*	**0.66**	**0.66**	NT	NT	NT	NT	1.33	1.50	NT	NT	1.66	2.66	1.50	8.00	2.66	4.00	NT	NT	NT	NT
*P. africanum*	NT	NT	2.00	0.83	NT	NT	NT	NT	1.66	1.66	NT	NT	NT	NT	NT	NT	1.00	**0.66**	2.00	2.00
*P. viridiflorum*	NT	NT	3.33	1.66	4.00	4.00	NT	NT	1.33	1.00	NT	NT	NT	NT	NT	NT	NT	NT	NT	NT
*P. africana*	NT	NT	1.33	**0.83**	NT	NT	NT	NT	**0.83**	**0.50**	NT	NT	NT	NT	NT	NT	1.66	**0.50**	1.33	1.00
*P. rostratus*	NT	NT	**0.50**	**0.50**	NT	NT	NT	NT	**0.25**	**0.20**	NT	NT	NT	NT	NT	NT	**0.66**	**0.83**	**0.66**	**0.41**
*R. melanophloeos*	NT	NT	**0.41**	**0.41**	**0.25**	**0.66**	NT	NT	**0.50**	**0.50**	NT	NT	NT	NT	NT	NT	**0.50**	**0.50**	**0.50**	**0.41**
*R. caffra*	NT	NT	3.33	4.00	**0.50**	1.00	NT	NT	1.66	1.66	NT	NT	NT	NT	NT	NT	NT	NT	NT	NT
*S. brachypetala*	1.00	**0.50**	NT	NT	NT	NT	**0.83**	**0.66**	NT	NT	1.00	**0.33**	**0.83**	**0.66**	**0.83**	1.33	NT	NT	NT	NT
*S. birrea*	1.00	**0.50**	NT	NT	NT	NT	**0.66**	**0.50**	NT	NT	1.00	**0.50**	**0.83**	2.00	**0.83**	2.00	NT	NT	NT	NT
*S. longepeduncu-lata*	NT	NT	8.00	2.00	4.00	1.33	NT	NT	1.33	1.66	NT	NT	NT	NT	NT	NT	NT	NT	NT	NT
*S. henningsii*	2.66	1.00	NT	NT	NT	NT	1.50	8.00	NT	NT	8.00	2.66	8.00	2.00	8.00	8.00	NT	NT	NT	NT
*S. cordatum*	NT	NT	1.66	2.66	NT	NT	NT	NT	2.00	3.33	NT	NT	NT	NT	NT	NT	1.00	**0.66**	1.66	2.66
*T. emetica*	3.33	1.66	NT	NT	NT	NT	**0.83**	1.66	NT	NT	5.33	1.66	1.66	1.66	3.33	8.00	NT	NT	NT	NT
*W. salutaris*	NT	NT	1.66	**0.50**	NT	NT	NT	NT	1.33	**0.25**	NT	NT	NT	NT	NT	NT	1.00	1.33	2.00	**0.41**
*V. karroo*	2.00	**0.66**	NT	NT	NT	NT	2.00	**0.66**	NT	NT	8.00	1.66	4.00	**1.00**	8.00	4.00	NT	NT	NT	NT
*V. robusta*	NT	NT	1.66	2.66	NT	NT	NT	NT	**0.83**	1.66	NT	NT	NT	NT	NT	NT	1.00	1.66	1.00	1.00
*Z. mucronata*	1.66	**0.66**	**0.50**	**0.83**	**0.50**	1.33	1.00	1.00	**0.83**	**0.83**	**0.66**	1.66	**0.25**	1.00	**0.50**	1.33	NT	NT	NT	NT
Ciprofloxacin positive control (µg/mL)	0.07		0.05		0.02		<0.02		<0.02		0.66		<0.02		0.07		0.03		0.05	
Acetone negative control	8.00		>8.00		>8.00		>8.00		>8.00		>8.00		>8.00		>8.00		>8.00		>8.00	
DMSO negative control	8.00		8.00		8.00		4.00		4.00		6.66		6.66		6.66		4.00		4.00	

* M = Methanol; ** DCM = dichloromethane; *** NT = not tested.

#### 2.2.3. *Schotia brachypetala*

Bark and roots of *S. brachypetala* are widely used in traditional medicine as a remedy for dysentery and diarrhea [13,14,43]. Noteworthy activity was observed against *E. coli* with the lowest MIC value of 0.33 mg/mL for the dichloromethane extract (Table 2). The dichloromethane extract from the present study was much more active against *E. coli*, in contrast to the ethanol bark extract with an MIC value of 3.31 mg/mL from an earlier study [94]. Methanol extract of the bark has also been screened against other *Shigella* species from other studies and demonstrated interesting activity against *Shigella dysenteriae* (MIC of 0.15 mg/mL) and against *Shigella flexneri* (MIC of 0.31 mg/mL) [16]. Organic extracts from the present study demonstrated noteworthy activity, which differed from results obtained by Van Vuuren and co-workers in 2015 [67]. The (methanol:dichloromethane) extracts from their study exhibited poor antimicrobial activity against *B. cereus*, *E. coli*, and *S. typhimurium* with MIC values of 8 mg/mL. In addition, corresponding activity was observed against *E. faecalis* with a MIC value of 0.63 mg/mL. The major reason for such variation in the MIC values could be attributed to the different choice of solvent extract [95]. Successful extraction of phytochemicals from plant materials may vary significantly depending on the type of solvent extract used [96].

#### 2.2.4. *Sclerocarya birrea*

The stem bark of *S. birrea* is widely used in traditional medicine for the treatment of stomach ailments, including diarrhea [14,40,50], gastritis, peptic ulcers, and stomach cancer [97]. Bark extracts demonstrated overall good antimicrobial activity against five bacterial strains screened with MIC values mostly ≤1 mg/mL (Table 2). Antimicrobial activity with a similar MIC value of 0.83 mg/mL against Gram-negative *S. typhimurium* and *S. sonnei* was recorded from the methanol extract. The dichloromethane extract exhibited similar activity with a MIC value of 0.50 mg/mL against *B. cereus*, *E. faecalis*, and *E. coli*. The results from this study differ with those obtained by Moyo et al. in 2011 [98] against *E. coli* (MIC of 3.12 mg/mL) reported from the dichloromethane extract of the twig bark from *S. birrea*. These differences in the MIC values may be attributed to different bacterial strains used and also to the age of the plant; thus, 83% of traders from other muthi markets [98] preferred the use of mature bark.

#### 2.2.5. *Pterocelastrus rostratus*

Bark decoctions are taken as emetics for respiratory ailments and administered orally for a blocked and wheezing chest [8,28]. Good antimicrobial activity was demonstrated from the extracts against the four bacterial strains tested with MIC values ranging from 0.20 mg/mL to 0.83 mg/mL (Table 2). Moderate activity against *K. pneumoniae* and *M. catarrhalis* with a MIC value of 0.66 mg/mL was noted from the methanol extract and 0.50 mg/mL against *S. aureus* from both extracts. Interesting activity with MIC values of 0.25 mg/mL (methanol) and 0.20 mg/mL (dichloromethane) was detected against *P. aeruginosa*. The broad-spectrum noteworthy antimicrobial activity of *P. rostratus* is not surprising, as *Pterocelastrus* bark species have been previously reported to be used for respiratory ailments in traditional medicine [43,46]. To the best of our knowledge, this is the first report on antimicrobial activity of the plant against respiratory tract pathogens.

#### 2.2.6. *Rapanea melanophloeos*

The bark is popularly used for skin, stomach, and respiratory tract infections (Table 1). Moderate activity against *S. aureus* with a MIC value of 0.41 mg/mL was recorded from the dichloromethane extract (Table 2). The lowest MIC value of 0.25 mg/mL against *S. epidermidis* was also demonstrated from the methanol extract (Table 2). In a study by Madikizela and co-workers [58], the dichloromethane leaf extract of *R. melanophloeos* exhibited weak activity against *S. aureus*, with a MIC value of 3.12 mg/mL. The bark of *R. melanophloeos* is used in many parts of sub-Saharan Africa for the treatment of respiratory tract infections [99]. Moderate activity of bark extracts with MIC values of 0.50 mg/mL was detected against *K. pneumoniae*, *M. catarrhalis*, and *P. aeruginosa*. The MIC value of 0.41 mg/mL was noted from the dichloromethane extract against *M. catarrhalis*. Acetone and water extracts of the bark were also screened against pathogens coincident with TB-related symptoms. The acetone extract exhibited a MIC value of 5 mg/mL, and water extracts displayed no activity against *Mycobacterium tuberculosis* [46,80]. Although the traditional use focused on sore throat and TB, the results presented here convey that *R. melanophloeos* could possibly be used for bronchial types of chest ailments, particularly if active compounds can be smoked or inhaled.

#### 2.2.7. *Warburgia salutaris*

The peppery scented bark is powdered and mixed with fats and applied on to the skin to treat infections, acne, and sores. The powdered bark is also smoked, sometimes mixed with the *Cannabis sativa* leaf, to treat colds and various chest complaints [13,14,39,40,42,43,71]. The dichloromethane extract inhibited *S. aureus* (0.5 mg/mL), *P. aeruginosa* (0.25), and *M. catarrhalis* (0.41 mg/mL), demonstrating non-discriminate inhibition across Gram-types. Further study conveyed that the drimane sesquiterpenes were important in achieving these effects [100].

### 2.3. Pharmacokinetics Perspective

The medicinal applications of bark in the present study generally require ingestion, topical application, or inhalation as smoke or vapor. For topical applications, the choice of extract solvent is relevant to the solubility of active compounds in the source material. Aqueous extracts will dissolve saponins, tannins, flavonoid glycosides, and so forth, which will be present in the methanol extracts that we made in the current study. Alternatively, fat extracts dissolve lipid soluble (lipophilic) ingredients, which will be present in the dichloromethane extract.

Due to topical application, direct contact-inhibition is all that is necessary to inhibit microbes associated with skin infection [101]. This means that transdermal penetration (or absorption) is not a limiting factor in achieving the higher MIC values demonstrated in the current study. There are many exceptions if consideration is given to subcutaneous infections, such as boils or acne, and in such cases lipophilic compounds are more suited to achieving these effects. However, saponins can deeply penetrate pores and the hair follicle bulb because of direct passage that is achieved by clearing lipophilic sebum (oil) due to the soap-like effects.

For gastrointestinal applications, in most cases the bark is boiled in water, and the aqueous solution is ingested. Although this conveys that aqueous solubility is an important precursor to achieve therapeutic effects, during the boiling process lipophilic ingredients are temporarily liquified and are driven into the infusion where they flocculate as the drink cools. The aqueous mixture is consumed with the flocculants, meaning lipophilic ingredients may also be participating or solely responsible for therapeutic outcomes. Nevertheless, as in the first example, contact-inhibition is all that is necessary to achieve bacterial inhibition [101].

For respiratory complaints that involve bronchial pathogens, there are two types of therapy that are enacted, with one involving inhalation of vapors or smoke, and the other involving oral ingestion, with the therapeutic effects expected to occur non-locally (or generally). In this latter case, the MIC values achieved in the current study are not low enough to be of any benefit, particularly because the systemic blood plasma concentration of these extracts (or active compounds) is expected to be in the order of hundreds of times lower than the MIC values in Table 2. However, many of the therapies are smoked or the emitted vapors inhaled, and, in these cases, merely contact inhibition is necessary to inhibit the pathogen. For example, *W. salutaris* is smoked, so the MIC values sufficiently validate that contact inhibition is the primary mechanism, and the effects are not necessarily enacted indirectly through the immune response [101].

### 2.4. Brine Shrimp Toxicity Assay

The % mortality of methanol and dichloromethane extracts from 31 medicinal barks induced in *Artemia nauplii* following 24 and 48 h of exposure, respectively, is shown in Table 3. All the tested medicinal barks showed less than 50% mortality except for the methanolic extract of *Trichilia emetica*, which exhibited 69.52% mortality at 48 h. The negative control demonstrated 0% mortality and, the solvent control demonstrated a maximum mortality rate of 5.34% after 48 h.

Differences in polarities of solvent extracts used for similar plant species will yield a diverse range of quantitative differences in phytochemicals, which may result in a different response to the brine shrimp assay. The methanol and dichloromethane extract of *A. adianthifolia*, *B. lucens*, and *T. emetica* displayed varying toxicity values. Bussmann et al. [102] also observed varying toxicity values between the same plant species due to the use of different solvent extracts. Despite these limitations, the results presented here seem to agree with other studies. Van Vuuren et al. [67], for example, also found that methanol and water extracts from the stem bark of *Warburgia salutaris* and *Syzygium cordatum* exhibited less than 50% mortality. Furthermore, the leaf methanol and water extracts of *Kigelia africana*, *Pittosporum viridiflorum*, *Schotia branchypetala*, *Sclerocarya birrea*, *Vachellia karroo*, and *Ziziphus mucronata* also demonstrated low toxicity in the brine shrimp assay, with some of these plants inducing mortality below 20% following 24 and 48 h of exposure [67].

The methanol bark extract of *Rauvolfia caffra* and *Kigelia africana* exhibited the lowest percentage mortality at 48 h against *Artemia franciscana*. Barks from plant species, including *Cinnamomum camphora*, *Dombeya rotundifolia*, *Harpephylllum caffrum*, *Ocotea bullata*, *Strychnos henningsii*, and *Sclerocarya birrea*, displayed minimal toxicity with less than 15% mortality after the 48 h exposure period.

The methanolic bark extract of *Trichilia emetica* displayed the highest level of toxicity, with 69.52% mortality, while the dichloromethane extract exhibited 19.60% mortality at 48 h (Table 3). These findings are in accordance with a study by Oryema et al. [103], who reported that methanol and water extracts of the roots were more toxic than ether extracts. The first report of toxic properties of *T. dregeana* (closely related to *T. emetica*) was observed in 1899, when an African woman died as a result of drinking a decoction of the bark for use as a laxative [104]. The bark of *T. emetica* is widely used in traditional medicine for treatment of gastrointestinal ailments [14,43]. The dichloromethane extract of the bark exhibited some degree of cytotoxicity in the monkey kidney cell with an ID_50_ of 50 µg/mL [105]. Furthermore, the root aqueous extract and ethyl ether fraction did not display toxicity (LC_50_ > 1000 µg/mL) in the brine shrimp bioassay [106]. To the best of our knowledge, this is the first report of brine shrimp lethality test results for the bark of *T. emetica*.

Both extracts from *Erythrina lysistemon* in this study did not exhibit toxicity against the brine shrimp (Table 3). A study by Prozesky et al. [105] also showed that acetone bark extract from *E. lysistemon* exhibited very low cytotoxicity when tested against the monkey kidney cell line.

Surprisingly, the bark of *Erythrophloeum lasianthum* appeared to be non-toxic in the brine shrimp assay. All parts of this plant are popularly known to be highly toxic. The active toxic principles include two diterpenoid alkaloids, cassaine and erythrophleine [28,40,41]. *Erythrophloeum lasianthum* is an important Zulu traditional medicine used as a snuff for treating headache [14].

The bark from *Erythrophloeum lasianthum* has been harvested for muthi market trade from as early as 1946 [107] as cited by Williams et al. [19], and it is still actively traded on the Johannesburg Muthi Markets. These findings are in agreement with Lewis [108], who also found that aqueous extract of the bark exhibited very low toxicity values in the brine shrimp assay. Aqueous extract of the pods and seeds, however, were slightly toxic. The mode of administration is a very critical aspect of traditional medicine that should be carefully handled, as there could be a thin line between toxicity and safety, depending on the dosage and method of preparation of the plant material. According to the information given by traders at the Johannesburg Muthi Markets, a pinch of finely powdered bark of *E. lasianthum* is taken as snuff for headaches but anything above that quantity results in severe bleeding from the nostrils and causes dizziness.

## 3. Materials and Methods

### 3.1. The Study Area: The Muthi Markets

#### 3.1.1. The Faraday Muthi Market

**Figure 4:**
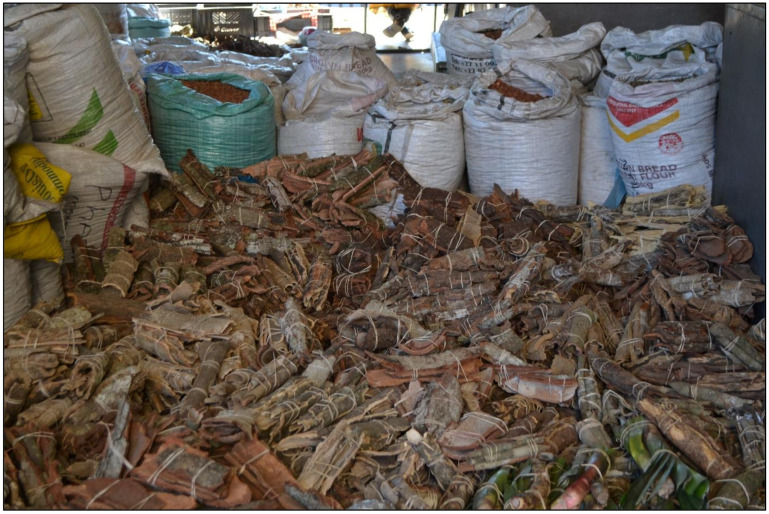
Typical stall at the Faraday Muthi Market, showing bark products. Photograph by E.L. Kotina.

The Faraday Muthi Market is located in Johannesburg, Gauteng Province (Figure 4). The Faraday Muthi Market has in the past decade become the biggest and most popularly known market in the southern African region and perhaps throughout Africa [109]. Furthermore, an estimate of 220 market traders were recorded at Faraday in October 2015, which decreased from over 300 traders recorded in October 2014 [109]. The trading stalls used for storing or packing plant products are usually outdoors, slightly exposed or covered, with very few traders that are found selling within the old and neglected public buildings [33]. There is also an active trade of animal products at the Faraday market such as snake and crocodile skin, and bones of carnivorous animals including lion, leopard, and different kinds of birds. These are usually sold as finely powdered material in very small amounts (teaspoon) and incorporated in plant mixtures to enhance the healing effect [23].

Purchasing of plant products from informal markets is a common day-to-day activity among the southern African population. The customers include local people, healers, and consumers from townships and rural and urban areas around Gauteng and other provinces, as well as neighboring countries such as Swaziland, Lesotho, and Mozambique. Most of these plant products are used to treat a variety of ailments including Alzheimer’s disease, sexually transmitted infections, cancer, malaria, inflammatory pain, and cardiovascular disorders [23]. Plants used for charm and magic are also popular, purchased most frequently for protection against enemies and to prevent or remove bad luck [13,23].

#### 3.1.2. The Kwa Mai-Mai Muthi Market

**Figure 5:**
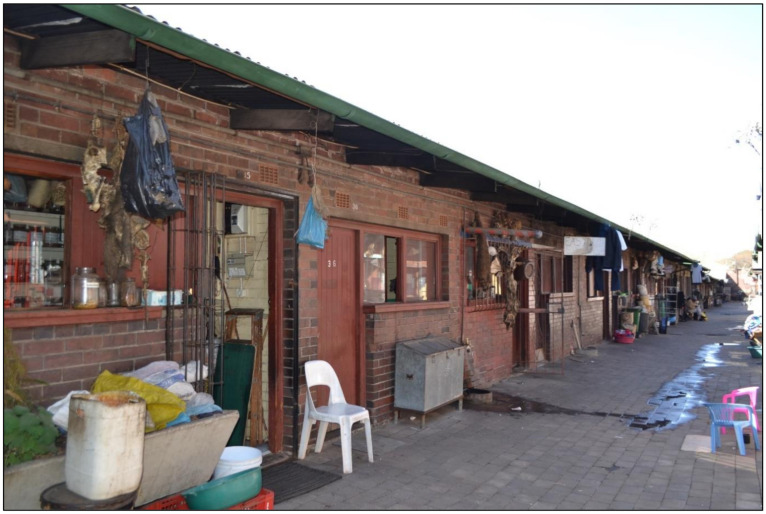
Typical selling stalls at the Kwa Mai-Mai Muthi Market. Photograph by E.L. Kotina.

The Kwa Mai-Mai Muthi Market is located south of Johannesburg under a bridge on the corner of Anderson and Berea Streets (Figure 5). The Kwa Mai-Mai Muthi Market is known as one of the oldest markets in Johannesburg, having opened about 50 years ago [23]. It is also known as “Ezinyangeni”, which means “the place of traditional healers”, as the majority of the traders are either traditional healers or “sangomas”. The city of Johannesburg municipality manages the muthi market. Unlike the Faraday Muthi Market, which sells mainly plant and animal products, the Kwa Mai-Mai Muthi Market has a wider diversity of products such as traditional attires, brooms that are often used in wedding ceremonies, and traditional mats (made from plant material). The trading stalls at the Kwa Mai-Mai Muthi Market are indoors and have private consultation rooms where customers can be offered “medical” service (Figure 5).

**Figure 6:**
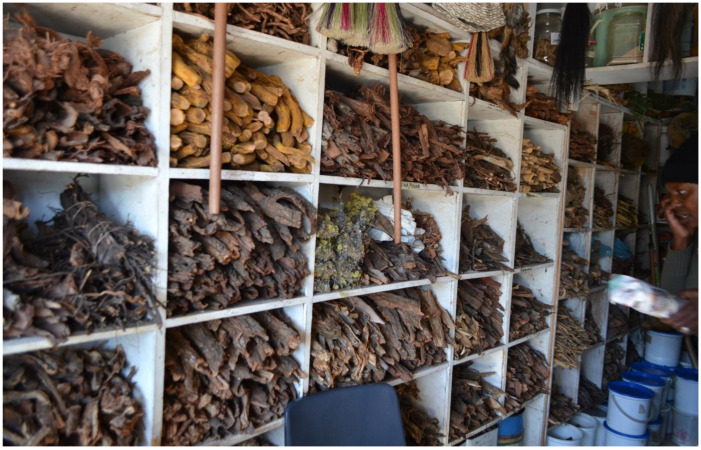
Inside the trading stall, illustrating the way in which bark is stored and dispensed. Photograph by E.L. Kotina.

There are various packaging and distribution methods adapted by traders when selling harvested bark material from the wild. These include raw, dried, solid pieces of material, or partially processed (chopped or pounded) products (Figure 4). Bark may be ground into fine powder, usually for use as snuffs or for incorporation into complex mixtures for use as insizi (powdered mixtures rubbed into scarification in the skin to alleviate pain). Packaging of the plant material is normally in the form of newspapers, recycled liquor bottles, or plastic bags (Figure 6). Medicines are commonly prepared with water as an extraction solvent to form a decoction or infusion.

#### 3.1.3. Trader Information and Data Recorded

The selection of traders was random and based on the willingness of an individual to participate, after clearly explaining in their home languages the purpose of conducting the research study. No formal interviews were constructed, as traders were giving out commercial information that a normal customer would require to use the product. For each package of bark sample purchased, the trader was asked the following information: (i) to give a vernacular name or names (“uyalazi igama lesintu lexolo”) to aid in the correct identification of the plant, (ii) main uses of the product (bark) (“lisetshenziselwani leli xolo”), (iii) method of preparation (“lisetshenziswa ngaluphi uhlobo”), and (iv) mode of administration (“lisebenza kanjani, uyaphuza, uyaphalaza noma uyachatha”). There were no ethical issues, since only commercial information of consumer and trader was recorded. No bark samples were collected from nature. One sample (*Warburgia salutaris*) was collected from a cultivated garden tree with permission from the owner (Prof van Wyk). Therefore, no permits were necessary. Plant identification was verified by a botanist, Prof B.-E van Wyk and Dr. E.L. Kotina, a bark anatomist from the University of Johannesburg. Voucher specimens have been deposited at the University of Johannesburg Herbarium. Bark samples often vary considerably, depending on the provenance or the age of the trunk from which it was sampled. This variability may result in errors and misidentifications when bark is described, and it therefore seems very difficult to create a reliable key for the identification of bark samples. In this study, there were cases whereby several bark samples of the same species were purchased from different traders in order to get a more representative sample and to account for natural variation. As a first step towards an identification guide for commercially relevant medicinal barks of southern Africa, the outer and inner bark appearance descriptions were prepared for all 31 barks recorded in this study (Table 1, Column 1). For accurate color identification, the Munsell Soil Chart was used to record the color codes.

### 3.2. Preparation of Plant Extracts

Dried bark material was cleaned with a brush to remove lichens, moss, and sand or dust particles. Dried bark samples were ground into powder using a high-speed Fritsch Pulverisette grinder (Labotec). Approximately 28 g of each powdered bark material was soaked in 150 mL of either methanol or dichloromethane for 48 h at room temperature. Methanol was chosen as an ideal solvent because it is likely to extract a wider diversity of polar compounds than water. It is often suggested that water extracts should be used when studying traditional medicine, because infusions and especially decoctions are popular traditional dosage forms. However, bark often contains high amounts of triterpenoid glycosides (saponins) and other compounds that may act as emulsifying agents [101]. As a result, traditional decoctions will also contain non-polar compounds. Furthermore, powdered bark is often directly ingested by licking it from the hand (a custom known as “khotha” in Zulu). Dichloromethane was selected to extract non-polar components. Extracts were filtered using a Whatman No. 1 filter paper and left for evaporation in a fumehood. The dried samples were re-dissolved in acetone or dimethyl sulfoxide (DMSO) for antimicrobial and toxicity assays, respectively.

### 3.3. Test Pathogens

Bacterial test organisms were selected based on the conditions that barks are reported to treat, i.e., infections of the skin, stomach, and respiratory tract. The following were used as test organisms: *Bacillus cereus* ATCC 11175; *Enterococcus faecalis* ATCC 29121; *Escherichia coli* ATCC 8739; *Klebsiella pneumoniae* ATCC 13883; *Moraxella catarrhalis* ATCC 23246; *Pseudomonas aeruginosa* ATCC 743971; *Salmonella typhimurium* ATCC 14028; *Shigella sonnei* ATCC 9290; *Staphylococcus aureus* ATCC 25923; and *Staphylococcus epidermidis* ATCC 12228. Each bacterial culture was grown in Tryptone Soya broth (TSB) (Oxoid, Ltd., London, UK), for 18–24 h at 37 °C.

### 3.4. Antimicrobial Assay

A serial micro-dilution assay was used to determine the minimum inhibitory concentration (MIC) values of the bark extracts. The technique involves the use of a 96-well micro-titre plate and tetrazolium salts as an indicator of microbial growth [95]. Using aseptic manipulation, 100 µL of TSB was aliquoted into each well of a sterile 96 well micro-titre plate. A volume of 100 µL of bark extracts at a starting concentration of 32 mg/mL in acetone or DMSO, as well as positive and negative controls, were transferred to the first row of the micro-titre plate. Ciprofloxacin (starting concentration 0.01 mg/mL) was used as a positive control while acetone and 10% DMSO were used as negative controls. Serial dilutions were performed longitudinally by transferring 100 µL of the well content and subsequently diluting the extracts and controls with 50% each time. Crude extracts were initially tested at 8.00 mg/mL and serially diluted two-fold 0.06 mg/mL. A 100 µL of the sub-culture (bacteria) was added to all the wells of each micro-titre plate. The culture was first diluted in broth (0.5 McFarland standard) and diluted 1:100 to give a density of approximately 1 × 10^6^ colony forming units/mL (CFUs/mL). Each micro-titre plate was then sealed with a sterile adhesive seal and incubated at 37 °C for 24 h. To confirm the purity of the cultures used, each diluted pathogen-broth mixture was also streaked onto TSA and incubated overnight. After incubation, 40 µL of *p*-iodonitrotetrazolium (INT: Sigma Aldrich, Johannesburg, South Africa) dissolved in sterile water was added to all micro-titre plate wells and used as a microbial growth indicator. The tests were performed in triplicate, and the MIC values were averaged.

### 3.5. Brine Shrimp Toxicity Screening

#### 3.5.1. Sample Preparation

Dried powdered stem bark, approximately 1 g, was soaked in 2–3 mL of methanol and dichloromethane. After the solvent extracts had evaporated, the dried solid material was dissolved in 2% DMSO to obtain a concentration of 2 mg/mL. The brine shrimp lethality was tested at this concentration because of the estimated low dermal penetration of active ingredients.

#### 3.5.2. Cytotoxic Brine Shrimp Assay

A total of 31 medicinal barks were subject to toxicity screening against brine shrimps hatched in seawater solution [110]. The brine shrimp eggs were incubated at 25 °C for 24 h. A mass of 16 g sea salt was dissolved in 500 mL distilled water for the preparation of artificial seawater. A sealed conical-shaped plastic container was placed in an inverted position in an empty glass beaker. The dried brine shrimp (*Artemia franciscana*) eggs (1 g) were then added to the prepared artificial seawater on the inverted plastic bottle. A rotary pump was placed at the bottom of the container to create aeration, in the presence of a constant light source. This was done in order to mimic the natural seawater environment to ensure suitable conditions for the eggs to hatch. This mixture was then poured on the micro-titre well lid and placed upside down with full exposure to the light source to channel or promote accumulation of the nauplii in clusters. This was done to increase the number of brine shrimp for collecting (pipetting). Then, 48-well micro-titre plates were prepared by adding 400 μL of the salt-water solution containing about 40–60 live nauplii to each well along with 400 μL of the bark extract. The same volume of 400 μL was used for the addition of positive control, solvent control, and negative control separately with the brine shrimp eggs. The positive control used was 1.6 mg/mL potassium dichromate, a highly toxic, frequently used, and recommended reference compound for aquatic organisms. The solvent control was DMSO, which was kept at 2%. The number of dead brine shrimp was counted by viewing the plates under a light microscope at 40× magnification following 24 and 48 h of exposure to the test samples at room temperature. After counting at 48 h, a lethal dose of acetic acid (Saarchem; 100% (*v*/*v*); 50 μL) was added to each well, and then a final death count was undertaken to calculate the percentage mortality. A mortality percentage of 50% and above was regarded as toxic [102].

## 4. Conclusions

The predominant language spoken on the Faraday and Kwa Mai-Mai Muthi Markets in Johannesburg is *isiZulu*, and it appears that most of the traders have *isiZulu* as their home language. The dominant medicinal culture on the markets is also Zulu, and very few traditional medicines from other cultures are sold.

Individual species of bark are commonly used for the treatment of more than one ailment. Many of the popularly traded medicinal barks used against skin ailments are explained anecdotally to promote wound healing and achieve cosmetic effects, such as to remove pimples. Common gastrointestinal ailments were diarrhea and intestinal worms. Commonly treated respiratory tract ailments include coughs, tuberculosis, and colds. In general, the studied medicinal barks demonstrated good antimicrobial activity against dermatological, gastrointestinal, and respiratory tract pathogens thereby validating the traditional and commercial uses reported by the Johannesburg Muthi Market traders. Based on the toxicity results from this study, it can be concluded that the moderate activity exhibited by bark extracts is not strongly influenced by the presence or absence of toxic properties of the bark material, since extracts displayed mortality rates of less than 50%, which is regarded as non-toxic. These results support the safety and efficacy of traded medicinal barks from the informal muthi markets of South Africa. The most potent bark species were *E. transvaalense*, *E. lysistemon*, *Garcinia livingstonei*, *Pterocelastrus rostratus*, *Rapanea melanophloeos*, *S. brachypetala*, *Sclerocarya birrea*, and *Ziziphus mucronata.* Some of these species have already been examined for phytochemical composition, but the connection between chemistry and antimicrobial activity is not fully elucidated for all the species examined here. Hence, a follow-up bioactivity guide fractionation process may help to identify antimicrobial actives and create leads for the nutricosmetics industry [111].

## Figures and Tables

**Table 3:** Percentage mortality of *Artemia franciscana* tested against a selection of bark extracts from 31 tree species.

Plant Species	Solvent Extract	Percentage Mortality @ 24 h	Percentage Mortality @ 48 h
*Albizia adianthifolia*	Methanol	23.40	48.75
*Albizia adianthifolia*	DCM	4.00	6.87
*Bersama lucens*	Methanol	15.62	29.68
*Bersama lucens*	DCM	6.50	10.00
*Calodendrum capense*	Methanol	9.92	34.04
*Cinnamomum camphora*	Methanol	1.63	9.83
*Croton sylvaticus*	Methanol	13.64	32.71
*Cryptocarya latifolia*	DCM	18.17	30.88
*Dombeya rotundifolia*	DCM	7.84	7.84
*Ekebergia capensis*	Methanol	8.97	19.17
*Elaeodendron transvaalense*	DCM	22.37	48.00
*Erythrina lysistemon*	Methanol	26.51	37.05
*Erythrina lysistemon*	DCM	10.21	16.68
*Erythrophleum lasianthum*	Methanol	11.11	20.74
*Erythrophleum lasianthum*	DCM	11.34	18.55
*Garcinia livingstonei*	Methanol	19.12	46.85
*Harpephyllum caffrum*	Methanol	9.87	13.61
*Kigelia africana*	Methanol	3.25	6.00
*Ocotea bullata*	Methanol	8.85	12.19
*Peltophorum africana*	Methanol	20.40	25.85
*Pittosporum viridiflorum*	DCM	12.57	17.94
*Prunus africana*	DCM	22.00	32.00
*Pterocelastratus rostratus*	DCM	12.99	26.33
*Rapanea melanophloeos*	Methanol	14.42	23.09
*Rapanea melanophloeos*	DCM	11.84	17.33
*Rauvolfia caffra*	Methanol	3.25	4.01
*Schotia brachypetala*	DCM	24.00	36.10
*Sclerocarya birrea*	Methanol	6.38	12.46
*Securidaca longependunculata*	Methanol	18.33	30.89
*Strychnos henningsii*	Methanol	4.08	5.51
*Syzygium cordatum*	Methanol	8.22	20.00
*Trichilia emetica*	Methanol	40.95	69.52
*Trichilia emetica*	DCM	7.84	19.60
*Vachellia karroo*	Methanol	7.99	18.21
*Vachelia robusta*	Methanol	11.97	18.30
*Warburgia salutaris*	DCM	13.61	22.33
*Ziziphus mucronata*	DCM	8.33	15.18
CONTROLS			
Negative (salt water 32 mg/mL)		0	0
Negative (2% DMSO)		2.11	5.34
Positive control (1.6 mg/mL potassium dichromate)		94%	100%

## Data Availability

Not available.
